# Exploring the innovative application of cerium oxide nanoparticles for addressing oxidative stress in ovarian tissue regeneration

**DOI:** 10.1186/s13048-024-01566-2

**Published:** 2024-12-05

**Authors:** Maya Lakshmanan, Monika Saini, Manasa Nune

**Affiliations:** 1 Manipal Institute of Regenerative Medicine, Manipal Academy of Higher Education, Manipal, Karnataka 576104 India; 2 Department of Obstetrics and Gynaecology, All India Institute of Medical Sciences (AIIMS), Ansari Nagar, New Delhi, 110029 India

**Keywords:** Ovarian tissue engineering, Oxidative stress, Female infertility, Nanotechnology, Cerium oxide nanoparticles

## Abstract

The female reproductive system dysfunction considerably affects the overall health of women and children on a global scale. Over the decade, the incidence of reproductive disorders has become a significant source of suffering for women. Infertility in women may be caused by a range of acquired and congenital abnormalities. Ovaries play a central role in the female reproductive function. Any defect in the normal functioning of these endocrine organs causes health issues and reproductive challenges extending beyond infertility, as the hormones interact with other tissues and biological processes in the body. The complex pathophysiology of ovarian disorders makes it a multifactorial disease. The key etiological factors associated with the diseases include genetic factors, hormonal imbalance, environmental and lifestyle factors, inflammatory conditions, oxidative stress, autoimmune diseases, metabolic factors, and age. Oxidative stress is a major contributor to disease development and progression affecting the oocyte quality, fertilization, embryo development, and implantation. The choice of treatment for ovarian disorders varies among individuals and has associated complications. Reproductive tissue engineering holds great promise for overcoming the challenges associated with the current therapeutic approach to tissue regeneration. Furthermore, incorporating nanotechnology into tissue engineering could offer an efficient treatment strategy. This review provides an overview of incorporating antioxidant nanomaterials for engineering ovarian tissue to address the disease recurrence and associated pathophysiology. Cerium oxide nanoparticles (CeO_2_ NPs) are prioritized for evaluation primarily due to their antioxidant properties. In conclusion, the review explores the potential applications of CeO_2_ NPs for effective and clinically significant ovarian tissue regeneration.

**Graphical Abstract:**

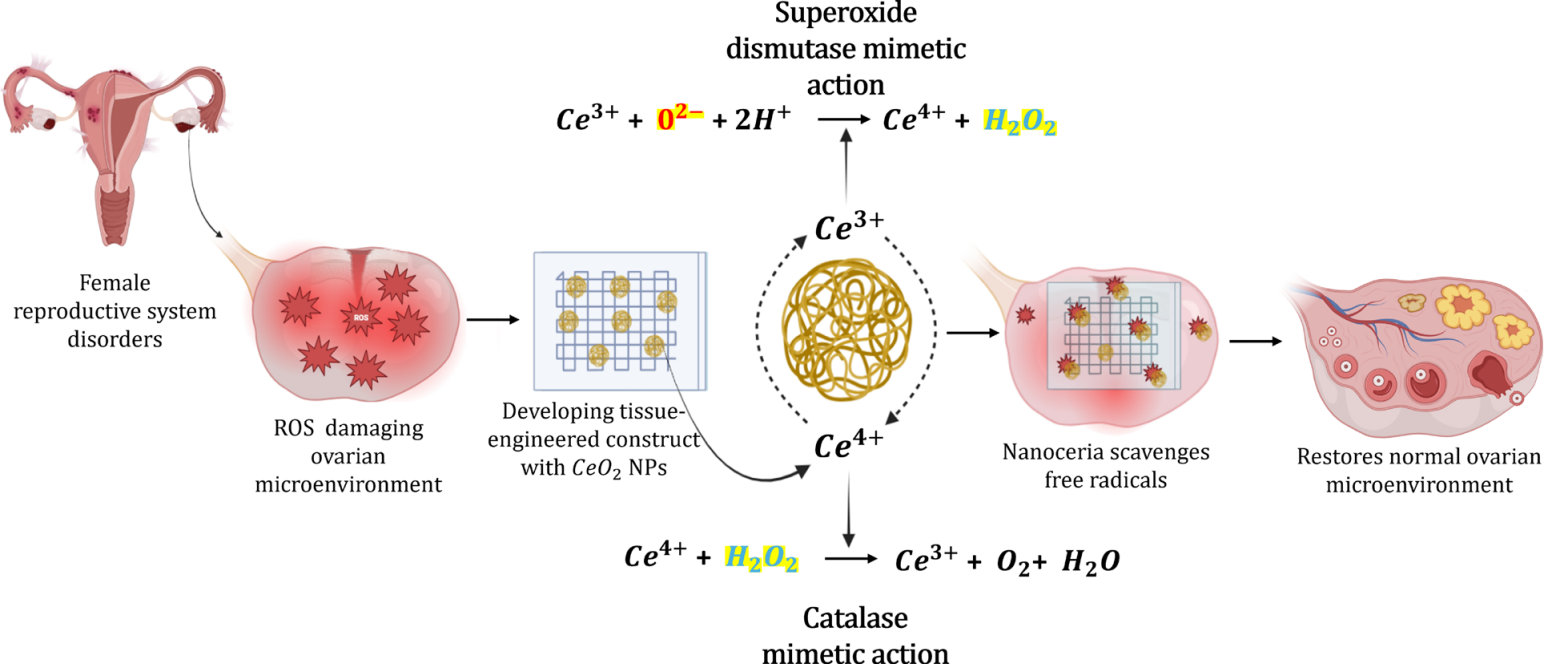

## Introduction

Infertility is a major health issue affecting millions of individuals of reproductive age worldwide. Infertility in women may be caused by a range of abnormalities in the ovary, uterus, fallopian tubes, and endocrine system along with general health conditions, inherited traits, lifestyle choices, and age [1]. A dynamic physiological interaction between the female reproductive organs and the complex interplay of hormones maintains the reproductive function in females by starting the menstrual cycle, production of ova, fertilization, zygote formation, supporting the growing fetus, maintaining pregnancy, and facilitating labor. Ovaries are fundamental to the reproductive function of females. They are the female gonads that control oogenesis and folliculogenesis. The ovaries cyclically produce gametes and secrete hormones such as estrogen and progesterone and control the hypothalamic-pituitary unit by negative and positive feedback mechanisms. These hormones play a major role in developing female characteristics as they interact with other tissues and biological systems in the body and have a key role in reproduction and maintaining pregnancy [2]. The ovarian somatic cells like theca cells, granulosa cells, and stromal cells interact with the surrounding ovarian extracellular matrix (ECM) and release hormones necessary for the maturation of the oocytes within the follicles, resulting in ovulation and later formation of corpus luteum essential for maintaining pregnancy after fertilization [3]. The ovarian microenvironment plays a critical role in the functioning of this endocrine gland and the ovarian ECM undergoes continuous remodeling and wound healing throughout folliculogenesis [4].

Any disruptions in the normal physiological functioning of the ovary will cause psychological and medical challenges in women. This would further affect the quality of life for women and ultimately result in infertility. The female infertility is broken down into uterine abnormalities, tubal obstructions, peritoneal factors, and ovulation disorders [5]. The ovulation disorders caused by disturbances in hormone production and menstrual cycle are common causes of infertility in women. WHO categorizes ovulatory disorders into 3 groups: Group I is hypothalamic-pituitary failure, Group II is hypothalamic-pituitary-ovarian axis dysfunction, and Group III is ovarian failure [6]. The complex molecular-cellular dynamics and unique microarchitecture of ovarian tissue are highly important for normal reproductive function [7]. This complexity is reflected in most of the ovarian diseases and female infertility. A significant amount of effort is required to restore fertility since gynecological disorders are poorly understood. The techniques that are currently used, even though they are effective, have difficulties at a wider application level and fail to restore the function of the tissue. Therefore, tissue engineering-based approach is observed to be an innovative alternative for the preservation of germ cells and restoration of ovarian function [8–10]. Recently numerous efforts in tissue engineering focused on mimicking ovarian tissue have been made, however, this field remains very less explored [11, 12]. The present review focuses on exploring the combination of nanoparticles with tissue engineering for effective tissue regeneration.

Nanotechnology is an interdisciplinary field of science involving designing and synthesizing nanoparticles ranging from 1 to 100 nm in size. These particles at the nanoscale possess a high level of functional specificity, as the chemical, physical, and biological attributes of the material are significantly different from their bulk solid matter. Irrespective of the physicochemical characteristics including size, shape, structure, composition, aggregation, and intrinsic sensitivity of cells, the high surface area and small size enable them to interact with tissue and cells at subcellular levels. Nanoparticles are successfully used in various applications like tissue engineering [13–15], gene [16], and drug delivery systems [17, 18], labeling and tracing proteins [19, 20], developing nano biosensors for detecting ROS [21], molecular imaging [22], diagnostics [23], and cancer therapy [24–26]. In recent years there has been a revolutionary advancement in integrating nanotechnology into regenerative medicine applications [15, 27]. This approach has further transformed the conventional regenerative medicine and tissue engineering approach to develop a more efficient and intricate system. The regulation of both biological and physical characteristics of nanomaterials along with their biocompatibility with a diverse range of cell types enables the utilization of nanotechnology to significantly impact tissue engineering applications [28, 29].

The applicability of nanotechnology with reproductive tissue engineering needs further research. Nanotechnology has been used for the diagnosis, drug delivery, and treatment of reproductive disorders. However, there is very little information on the use of nanotechnology in reproductive tissue regeneration. In this review, we discuss the possibility of using the most abundant rare earth metal oxide-based inorganic nanoparticle, cerium oxide nanoparticle (CeO_2_ NPs/nanoceria) for ovarian tissue engineering. Metal oxide nanoparticles possess distinct properties and CeO_2_ NPs have gained much attention in recent years due to their extensive range of biomedical applications. The versatility and functionality of CeO_2_ NPs make them a promising material in a wide range of applications from medicine to industry. They possess unique physical and chemical properties at the nanoscale due to their large surface area and high reactivity. CeO_2_ NPs display remarkable multifunctional properties including antioxidant, anti-apoptotic, anti-inflammatory, anticancer, and anti-bacterial and it has both angiogenesis-inhibiting and activating properties at varying concentrations making it a versatile material [30–32]. The redox cycling and self-regenerating properties enable CeO_2_ NPs to scavenge free radicals and protect against oxidative damage, making them a promising candidate for biomedical applications. Reproductive system disorders at present have a higher mortality rate due to poor diagnosis. The current treatment options include invasive surgeries, hormone therapy, anti-inflammatory drugs, and chemotherapy which are often accompanied by significant limitations including incomplete treatment, a high chance for disease recurrence, side effects potentially impacting fertility [33]. From the prepubertal to post-menopausal stage the diseases develop in females regardless of their age. Hence there is a need for improved treatment options and nanotechnology offers a promising approach to address these challenges, by not only enhancing treatment efficiency but also promoting tissue regeneration for improved fertility outcomes. The review investigates the innovative and potential use of combining CeO_2_ NPs with a tissue engineering-based approach for regenerating ovarian tissue, thereby restoring reproductive function and potentially reducing disease pathophysiology in females.

## Anatomy and physiology of ovary

**Fig. 1:**
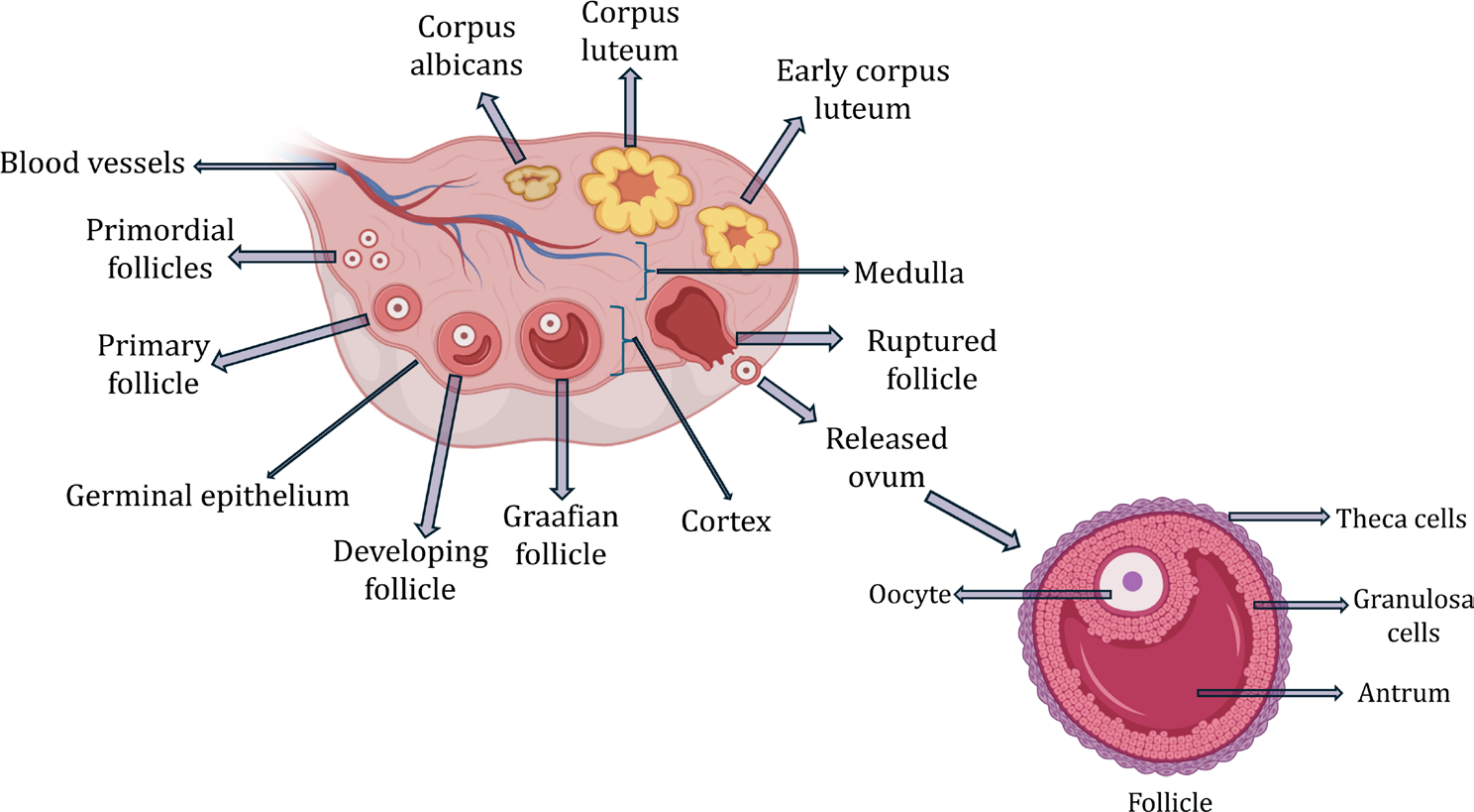
Anatomy of ovary and follicle

Ovaries are small oval-shaped glands attached by ovarian ligaments on either side of the uterus against the pelvic wall in the ovarian fossa. The suspensory ligaments attach them to the pelvic wall. Each ovary is covered by an outer layer of simple cuboidal ovarian epithelium and underneath this layer is tunica albuginea, a dense connective tissue capsule. The ovary is further compartmentalized into an inner medulla with loose connective tissue which is highly vascular, and a dense and granular outer cortex comprised of germ cells (oocyte) and somatic cells (stromal cells, granulosa cells, and theca cells) (Fig. 1). Follicles are the structural and functional units of ovary consisting of the oocyte which is surrounded by granulosa and theca cells along with different types of ECM including the basement membrane, zona pellucida, and antrum [34–36]. The ovarian reserve at the birth of females ranges from one to two million, however, most of the follicles undergo atresia from birth to puberty where approximately 400,000 to 500,000 remain, and only less than 1% is used until the menopause stage. The rest of the follicles do not undergo maturation and are degenerated and there is a gradual decline in follicles and periodic release of oocytes. The interaction of oocytes with granulosa cells is crucial for the maturation and growth of female gametes [37–39]. The ovarian ECM is involved in maintaining the function of several cell types in the ovary. It is essential for the proliferation of granulosa cells, and stimulation of the cumulus cells to release cytokines and have a direct influence on oocyte maturation and ovulation. The mechanical properties of ECM influence ovarian physiology and play a crucial role in folliculogenesis, steroidogenesis, hormone secretion, growth factor distribution, and the ovarian intracytoplasmic processes ultimately impacting oocyte maturation [40]. The ovarian ECM undergoes continuous remodeling and wound healing throughout folliculogenesis where there is a differential synthesis of ECM components and interaction with various growth factors, cytokines, metalloproteinases, and enzymes that maintain the balance between ECM degradation and synthesis [41]. The proteolytic action of enzymes and molecules on ECM proteins is essential during ovulation where collagen fragmentation occurs along with basement membrane breakdown for cumulus expansion accompanied by luteinizing hormone (LH) secretion for the extrusion of cumulus oocyte complex. The composition of ECM changes significantly as the follicle matures [3, 42, 43].

Ovaries are the endocrine organs of the female reproductive system that maintain the reproductive function by controlling oogenesis and folliculogenesis. They consist of small fluid-filled sacs called follicles at different stages of development. The immature oocytes undergo maturation and periodic release after the onset of puberty and have a potential chance to get fertilized [34]. The ovarian function is controlled by Gonadotropin-releasing hormone (GnRH) released from the hypothalamus, which stimulates the pituitary glands to release LH and follicle-stimulating hormones (FSH) which in turn control the menstrual cycle [2]. The 3D architecture of follicles with surrounding granulosa cells and theca cells plays a vital role in the maturation of oocytes. They are sites for steroid production and the action of gonadotropins. The granulosa cells line inside of the follicle surround the oocyte and produce estrogen in response to FSH. Theca cells, located in the outer layer surrounding granulosa cells, produce androgens in response to LH. After the onset of puberty FSH released by the pituitary gland stimulates follicle development and in turn, stimulates the release of estrogen. The increase in estrogen triggers the mid-cycle surge in a secondary pituitary hormone, LH which leads to ovulation by rupture of the follicle. Once the oocyte is released, the ruptured follicle forms the corpus luteum, an endocrine structure that produces progesterone which prepares the endometrium for potential implantation and supports pregnancy [34, 44]. Once ovulation occurs, the oocyte may get fertilized in the fallopian tube, develop into a zygote, and travel down the fallopian tube to get implanted into the thickened uterus and continue developing. If fertilization does not occur, the corpus luteum degenerates and progesterone levels come down triggering menstruation [4, 45].

## Prominent ovarian diseases

**Fig. 2:**
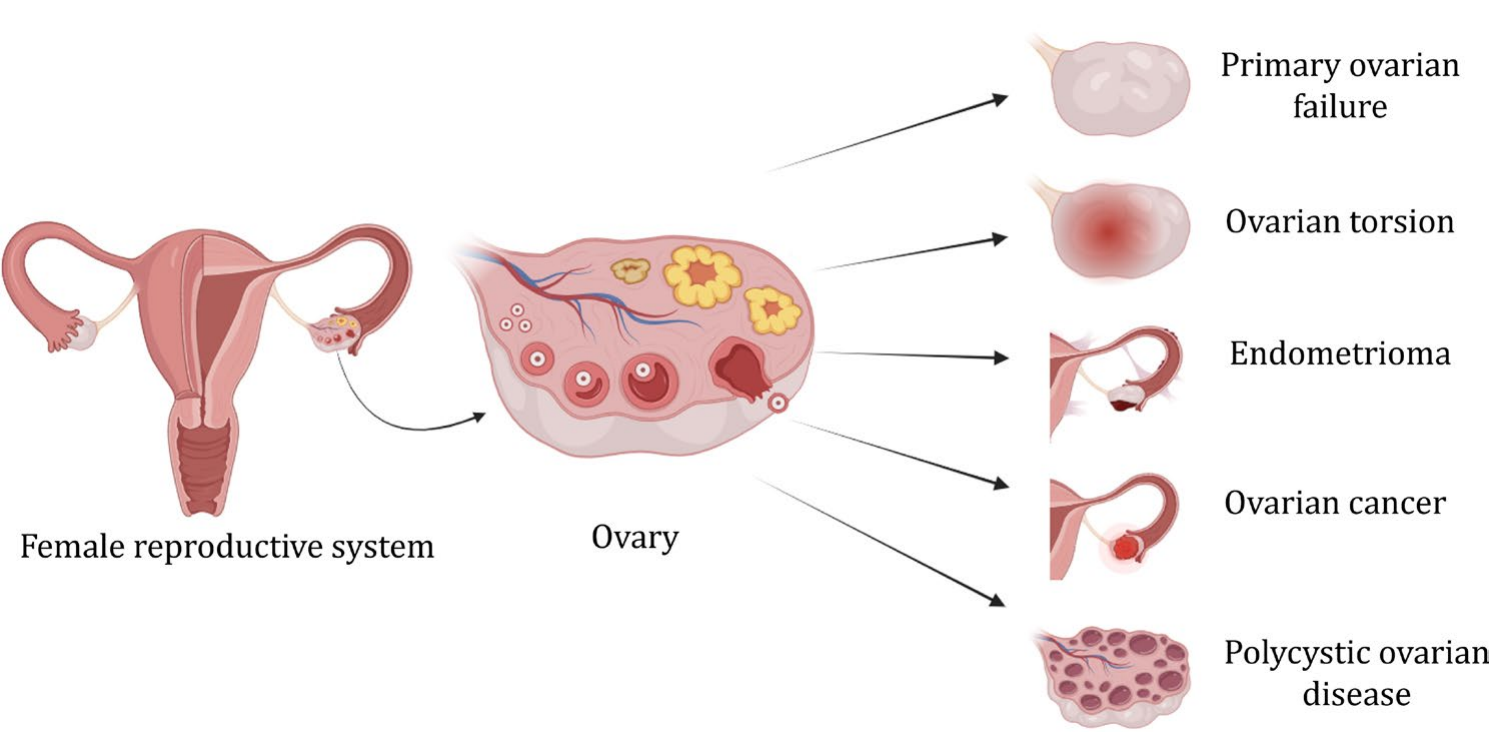
Prominent ovarian diseases

Ovaries play a key role in the functioning of the female reproductive system. Ovarian dysfunction can lead to a range of psychological and medical issues, extending beyond infertility, as the hormone interacts with other tissues in the body. The common diseases affecting the ovary include endometriosis, primary ovarian failure, ovarian torsion, ovarian cancer, and polycystic ovarian disease (Fig. 2). The incidence of female reproductive system diseases has shown an increasing trend over the years and is particularly observed in younger girls. These diseases can affect the female reproductive system causing damage to the organs, hindering their normal function, and potentially causing infertility. This negatively impacts the physical and mental well-being of the patient and their families. Some of the most common ovarian disorders affecting the female reproductive system are summarized as follows:

### Premature ovarian failure

Premature ovarian failure (POF) or Primary ovarian insufficiency (POI) is a prominent, recurring clinical syndrome impacting approximately 1–5% of the female population before 40 years of age [46]. It is characterized by cessation of normal function of ovaries, absence of menarche because of reduced ovarian reserve, hypergonadotropic hypogonadism with abnormally increased levels of FSH, and decreased levels of estrogen [47]. It is non-physiological amenorrhea or oligomenorrhea in females after puberty for at least 4 months, with poor ovarian response to circulating hormones, and absence of folliculogenesis before the age of 40 [48]. Even though the exact etiology of POI is not clear, it can be iatrogenic or idiopathic, particularly linked to cancer-induced oophorectomy and chemo- or radiation therapy, genetic factors, chromosomal defects like Turner syndrome, autoimmune and metabolic disorders, infections, physiological stress, and oxidative stress [49]. It is a major long-term side effect in young cancer patients after chemotherapy. It impacts the quality of life for women as the elevated gonadotropins and lower levels of estrogen can cause menopausal symptoms like hot flashes, insomnia, and night sweats [50]. This imbalance in hormones increases the chances of cardiovascular diseases, osteoporosis, and diabetes mellitus with disturbed menstrual cycles. POF causes infertility and compromised mental health in women [51].

### Ovarian torsion

Ovarian torsion or adnexal torsion is a medical emergency where the fallopian tube or the ovary twists around the supporting ligaments completely or partially and cuts off blood supply to the ovary [52]. It is a condition associated with high morbidity and low prevalence rates due to delayed diagnosis. The torsion can occur in females irrespective of age and even though very rare in pediatric patients, it is challenging to diagnose [53]. The incidence of ovarian torsion is commonly observed in women of reproductive age, during pregnancy, and post-menopausal women [54]. The presence of ovarian cysts and tumors increases the risk of ovarian torsion. The ligament connecting the ovary and uterus has vascular flow from both the organs and the movement and rotation of the ovary in the ligaments further obstruct the vasculature causing ischemia. An enlarged ovary tends to rotate on its axis and twisting of the ligaments causes compression of arteries and blocks the blood supply to the ovary. There is a high chance of internal hemorrhage in case of a ruptured ovarian mass and cyst [55]. If the blood supply is blocked for a longer time, it could damage the ovarian tissue and affect the normal functioning of the ovary, causing inflammation, potential tissue necrosis, hormonal imbalance, and infertility [56, 57].

### Endometrioma

Endometriosis is a common disease in women of reproductive age affecting 7–10% of the general female population. It is an estrogen-dependent inflammatory disease, characterized by the histological presence of benign functional endometrial glands or stroma outside the uterine cavity [58]. Approximately, 25–50% of infertile women have endometriosis and 30–50% of women with endometriosis are infertile [59]. Ovarian endometrioma is a major subtype of endometriosis, found in up to 17–44% of women with endometriosis [60]. Endometrioma is one of the most frequent adnexal masses in the premenopausal population characterized by the presence of menstrual fluid-filled chocolate cyst [61, 62]. There are many hypotheses proposed to date regarding the pathogenesis of endometriosis like the theory of retrograde menstruation, stem cell theory, theory of coelomic metaplasia, Mullerian embryonic remnant abnormality, theory of lymphatic and vascular metaplasia [58]. The pathophysiology of the disease is linked to hormonal factors like estrogen dependence and progesterone resistance; genetic factors; an increase in inflammatory mediators, angiogenesis, and oxidative stress due to increased Reactive Oxygen Species (ROS) leading to lesion formation, making it a complex disease [63, 64]. Endometriosis negatively impacts the ovarian reserve by exposing healthy ovarian tissue to free radicals, which reduces the pool of primordial follicles, and further affecting the quality and quantity of oocytes and embryos [65]. Endometriosis is usually underdiagnosed and when the endometriomas are left untreated for a period it is likely to have ovaries enlarged in size and can damage the ovarian cortex affecting the process of ovulation [66]. The delayed diagnosis directly affects ovarian reserve by age-related factors along with the oxidative damage of the follicle surrounding the endometrioma and it happens much faster than natural decline [67]. Studies also reveal that the progression of endometrioma can result in fibrosis and smooth muscle metaplasia in the cortex and reduce follicular reserve and quality of oocytes [68]. Even though the malignant transformation of endometrioma is rare, the risk factor still exists and needs to be considered seriously [69, 70].

### Ovarian cancer

Ovarian cancer accounts for the most common, recurrent, and fatal gynecological cancer in women, and children are frequently diagnosed late due to a lack of effective screening and delayed onset of symptoms [71]. Even though the prevalence rate of ovarian cancer is less, it is associated with a high mortality and morbidity rate compared to other gynecological tumors [72]. Although the advancements in therapeutics and diagnostics have improved leading to an increase in the survival rate of cancer patients, they are more likely to be infertile. The underlying cause of ovarian cancer is still unclear, but it originates at the distal end of the fallopian tube and spreads to the ovary [73]. The occurrence of ovarian tumors is uncommon in children but are observed to be highly aggressive malignant tumors. Young pre-pubertal and pubertal children have a frequency of germ-cell malignant tumors and functional or benign cysts with ovarian torsion [74]. The other neoplasms can also be detrimental to oocyte and ovarian tissue causing ovarian failure, and early menopause resulting in infertility due to the high levels of radiation and chemotherapy both in adult and young patients [75]. Ovarian cancer can be grouped into stromal, sex cord, germ cell, and epithelial cancer based on their place of origin. Epithelial ovarian cancer (EOC) is the most recurrent and fatal one [76, 77]. WHO classifies the EOC into subtypes such as low-grade serous, high-grade serous, clear cell, endometroid, and mucinous carcinoma [78]. The development and progression of cancer depends on ovarian cancer microenvironment, which includes the immune cells that often promote metastasis and tumor growth. Additionally, cancer-associated fibroblasts alter the ECM composition, growth factors, and cytokines and enhance tumor proliferation, invasion, and migration. This further modulates the immune response and angiogenesis providing a compatible niche for supporting the tumor [79]. There is a dynamic interplay of molecular and cellular components which further impacts the ovarian follicular reserve. The malignancies have deleterious effects on the germ cells and their development. They have significantly reduced hormones and morphological defects in the gametes which further impacts the fertility outcome [78, 80]. The iatrogenic conditions due to radiation therapy and chemotherapy further cause gonadal damage, disrupting hormone secretion, and follicular development resulting in infertility [70, 81].

### Polycystic ovarian syndrome

Polycystic ovarian syndrome (PCOS) is the most prevalent inflammatory endocrine disease with lifelong health effects, particularly in 4–12% of reproductive-age women. The pathophysiology of PCOS is complex and heterogeneous [82]. They are associated with an imbalance in the gonadotropin-releasing hormone (GnRH) with aberrant gonadotropin secretion, hyperandrogenism, ovulatory disorder, and insulin resistance in women. The hypothalamus-pituitary-ovarian axis is disrupted causing increased sensitivity of pituitary GnRH leading to abnormalities in ovarian functions. There is enhanced secretion of androgen which directly impacts ovulation and follicular maturation [83]. The ovaries tend to be in an enlarged polycystic stage with multiple small antral follicles resulting in excess anti-mullerian hormone but with reduced primordial follicles. These excess small follicles disrupt the normal ovarian microenvironment causing hormonal disruptions by altering the positive negative feedback mechanism of progesterone and estrogen [84]. PCOS is associated with excess LH which further causes theca cells to secrete excess androgen. This hyper secreted androgen is responsible for insulin resistance in PCOS patients. This leads to hyperinsulinemia and increased secretion of androgens [85]. PCOS is associated with an inflammatory ovarian microenvironment with increased oxidative and endoplasmic reticulum stress aggravating cell apoptosis and follicular atresia. Even though multiple small follicles are present in polycystic ovary, there are chances of oocyte maturation arrest with granulosa cell disruptions and anovulation. There are consistently poor-quality oocytes with meiotic defects in these patients which significantly affects the fertilization potential [86]. PCOS pathogenesis is associated with environmental and lifestyle factors, genetic factors, and high heritability along with menstrual, metabolic, and endocrine dysfunction of the ovary [85]. PCOS increases the chance of endometrial cancer as amenorrhea develops hyperplastic endometrium. PCOS women have anovulatory cycles with irregular or heavy bleeding, hirsutism, and acne. The anovulation contributes to infertility. The reproductive dysfunction with metabolic disorders like obesity, cardiovascular disease, insulin resistance, hyperinsulinemia, impaired glucose metabolism, increased chances of diabetes, and liver diseases aggravates the disease condition [87].

## Oxidative stress and its association with ovarian diseases

**Fig. 3:**
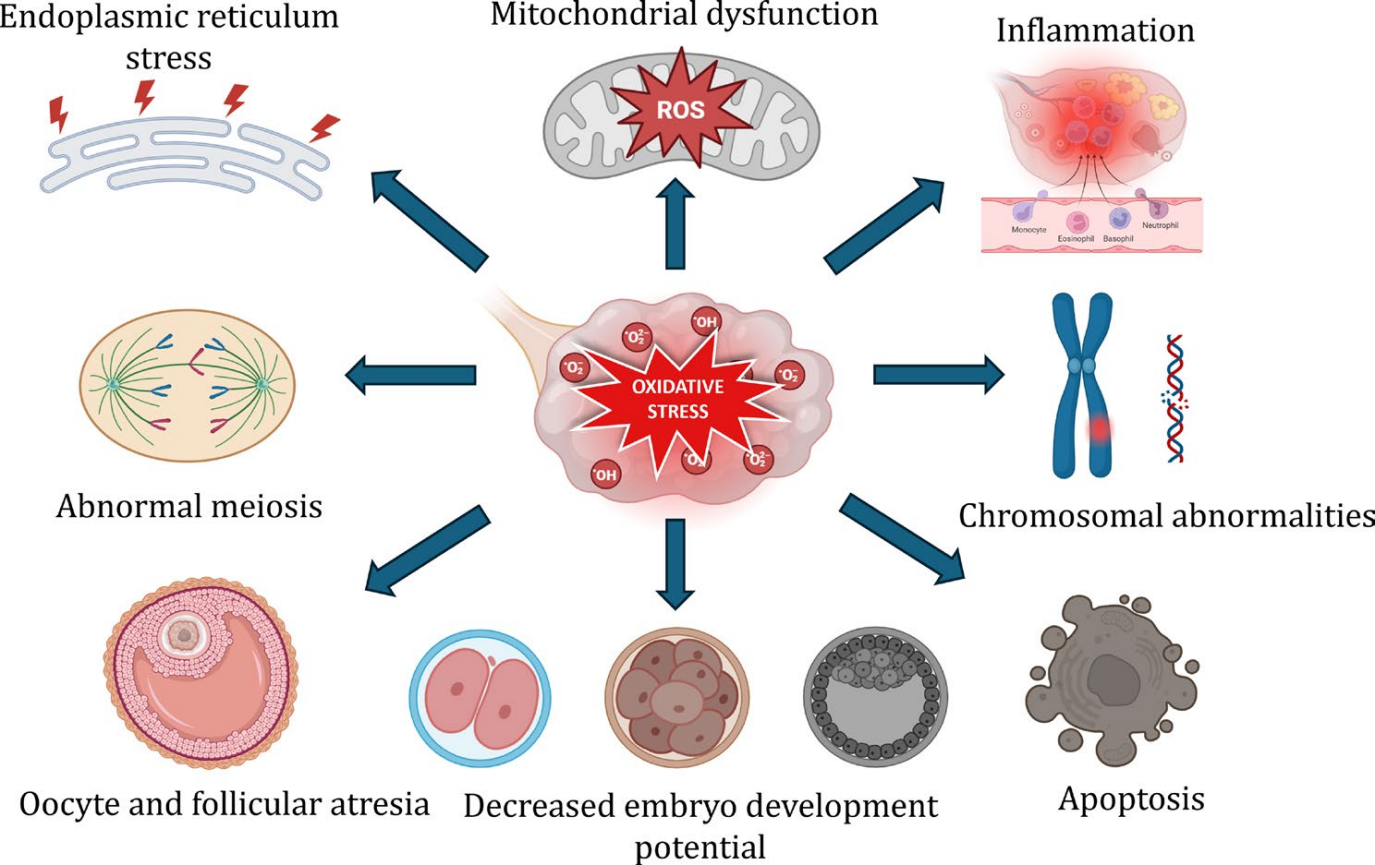
Role of ROS in disease pathophysiology

Even though the pathogenesis of ovarian diseases is not fully understood, oxidative stress is evidently observed in most pathological conditions (Fig. 3). They contribute to disease development and progression [88]. Oxidative stress occurs when the production of ROS surpasses the body’s antioxidant defense system to repair the damage [88]. This can potentially disrupt the normal cellular integrity and function. ROS includes oxygenated molecules, free radicals, and non-free radicals. Molecules like singlet oxygen, hydroxyl radicals, and hydrogen peroxide, superoxide radicals are common metabolic byproducts of cellular respiration. They contain oxygen atoms with strong oxidizing ability and are normally produced in the body. In the ovary, ROS is generated through multiple pathways and there is a balance in the antioxidant system and physiological level of ROS [89, 90]. At the physiological range, ROS is necessary for maintaining the proper functioning of the ovary and is essential for the normal ovarian cycle, meiotic division, ovulation, and maintenance of the corpus luteum. There is a complex interaction of antioxidants and ROS in the ovary [91]. A balanced antioxidant-ROS system maintains the meiotic arrest and its resumption. In the presence of ROS and deprived antioxidant production, every month the dominant oocyte completes meiosis [92]. ROS is essential for oocyte development, fertilization, and implantation. Ovulation is a critical process in the ovary resembling inflammation [88]. The surge in LH before ovulation increases inflammatory precursors in the ovary, leading to excessive ROS production. They are also involved in the expansion of cumulus cells, the production of progesterone by maintaining the corpus luteum, and activating signals for successful ovulation [93]. The oocytes are protected by the antioxidant system in the cumulus cells, granulosa cells, and follicular fluid at physiological conditions. The enzymatic and non-enzymatic antioxidant defense system timely removes the ROS and maintains intraovarian homeostasis [88, 94, 95].

Even though ROS is necessary for the normal physiological function of the ovary, the imbalance in ROS production and the antioxidant system is a major contributing factor to ovarian diseases [96]. At excess levels, ROS can impact the signaling pathway and cause abnormal outcomes resulting in various pathologies like ovarian cancer, PCOS, Endometrioma, POF, and age-associated ovarian damage [97]. Apart from this, lifestyle choices like smoking, drinking, a high-sugar diet, and environmental factors also contribute to ROS production and cellular senescence. In the presence of excess ROS, functional tissue repair and recovery is very difficult due to high cellular damage and inflammation. Hypersecretion of ROS leads to oxidative damage of genetic material, proteins, and lipids and induces oxidative stress. The abnormalities caused by oxidative damage are seen in most of the ovarian dysfunction [98, 99]. In various disease conditions, the ROS scavenging capacity is reduced, contributing to tissue damage. The mitochondria are the most abundant organelles in oocytes, and aerobic metabolism is the prime source of ROS production. The mitochondrial DNA is more vulnerable to oxidative damage which further disrupts the respiratory chain function [93]. Oxygen molecules are reduced to O_2_^**−**^ in the presence of electrons leaked from the mitochondrial respiratory chain. This primary ROS is converted to secondary ROS like H_2_O_2_, OH^**−**^ through catalytic reactions [89, 100].

Ovarian aging and premature ovarian failure are associated with mitochondrial dysfunction [101]. The reduced copy of mitochondrial DNA in most ovarian dysfunction, consequently, decreases the fertilization potential of oocytes and notably impacts the embryo quality [102]. The endoplasmic reticulum (ER) is another site of ROS production. The protein synthesis is affected during increased ROS in ER causing ER stress. The enhanced proinflammatory cytokines due to activation of lipid peroxidation during oxidative stress further impact the oocyte cell membrane [103]. Oxidative stress derails the normal functioning of proteins in oocytes and follicles by oxidizing the amino acids in proteins. The function and enzymatic activity of protein are lost and the disrupted proteolytic cleavage results in the accumulation of oxidized amino acids [104]. Even though the cellular antioxidant system defenses against oxidative damage by ROS, oxidative stress is a major cause of telomere shortening. Excessive shortening affects chromosome stability and genomic integrity [105]. POF is associated with reduced telomerase activity and shorter telomere length, which is positively associated with the oocyte and embryo quality [106].

Oxidative stress and inflammation are strongly associated with promoting chronic diseases. Elevated inflammatory markers are linked to an increased risk of ovarian aging and early menopause [88]. The critical role of oxidative stress in ovarian endometrioma suggests reduced oocyte quality and fertilization rates. The endometriotic ovary is observed to have an increased inflammatory response affecting the surrounding stroma. Endometriotic cyst increases ROS production, causing additional damage to the surrounding tissue and trigger autophagy and apoptosis in oocytes. The prolonged imbalance in ROS and antioxidants stands out as a major factor in the malignant transformation of endometrioma [64]. ROS contributes to tumor development and metastasis by promoting angiogenesis and DNA mutations. Alternatively, elevated ROS can induce tumor apoptosis [107]. Pathogenesis of PCOS is poorly understood but ROS plays a critical role in aggravating the endocrine disorder. Studies reveal the elevated oxidative stress markers in PCOS patients and their significant correlation with obesity, insulin resistance, chronic inflammation, and hyperandrogenemia [108]. Altered oxidative stress in PCOS has a detrimental impact on the ovarian microenvironment, resulting in anovulation and infertility which carry lifelong health consequences [109].

The interaction between ROS and biological molecules activates cell apoptosis and damages the ovarian cortex. This reduces the quality of oocytes by causing chromosomal instability, and meiotic abnormalities, and interferes with nuclear and cytoplasmic maturation, particularly in spindle formation [62, 110]. Oxidative stress can interrupt communication between oocytes and follicles leading to ovulatory defects. ROS can decrease the fertilization rate of oocytes and increase the likelihood of aneuploidy, DNA fragmentation, and induce apoptosis. Apoptosis of oocytes can directly damage the germ cells while granulosa cells apoptosis deprives the ovarian cells of nutrition leading to metabolic disorders, ultimately resulting in organ failure.

## Current treatment approaches and associated constraints

The choice of treatment for ovarian disorders varies among individuals based on factors such as type and severity of symptoms, age, fertility, and treatment goals. Ovarian diseases are associated with idiopathic, iatrogenic, hormonal imbalance, and oxidative stress-related etiology and are commonly underdiagnosed and this delay in diagnosis leads to further complications [66]. Nonetheless, these therapies are palliative, although they can improve the quality of life for women, they are incapable of restoring normal ovarian functions like folliculogenesis and steroidogenesis.

### Surgical interventions

The conventional treatment option for ovarian endometrioma and cysts involves surgical techniques. Advanced surgical skills are required for the excision of endometrioma and cyst because there is a significant risk of damaging ovarian reserve post-cystectomy. Repeated surgery does not improve fertility outcomes and often damages the ovarian tissue and injures the ovarian vasculature. Studies have demonstrated reduced follicular count after cystectomy and have been questioned concerning damage to the operated ovary [111, 112]. Laparoscopic cystectomy is the first line of treatment for endometrioma and has reported an increased risk of ovarian damage and significantly lower antral follicular count and anti-mullerian hormone levels post-surgery [113, 114]. There is a meta-analysis on the high chance of cycle cancellation with a reduction in response by the ovary to ovarian stimulation and low oocyte quality for women with endometrioma [66]. There is no evidence that cystectomy can improve the success rate of artificial reproductive techniques (ART) [115, 116]. Oophorectomy is preferred during severe cases, especially in patients with ovarian cancer. Removal of the ovary, which is an endocrine organ results in long-term hormonal imbalances and infertility. Drawbacks of surgery include the formation of postoperative adhesions and the possibility of incomplete disease removal. It was observed that the ovarian reserve was significantly reduced. Sometimes a hysterectomy is performed with salpingo-oophorectomy to minimize the risk of malignancy spreading to the ovary, further affecting the reproductive function in females [117]. In the case of ovarian torsion, the treatment is preferably surgical detorsion, a laparoscopy is done to evaluate the condition of the ovary, and an attempt will be made to untwist it. If the ovary is observed to have undergone necrosis due to a prolonged block of blood supply, an oophorectomy will be done to remove the ovary [118]. A salpingo-oophorectomy is sometimes performed if both the fallopian tube and ovary are not viable to reduce the recurrence of the condition. Prolonged diagnosis can cause serious tissue damage and affect fertility. There is also an enhanced risk of post-operative infection due to necrotic tissue [52].

### Artificial reproductive techniques

Artificial reproductive techniques (ART) like In vitro fertilization (IVF) and Intra cytoplasmic sperm injection (ICSI) are commonly applied for sub-fertile and infertile women to conceive, however, studies have reported the negative impact of endometrioma on the quality and quantity of oocyte and embryo and implantation rate [59, 119]. Recent studies also demonstrated a significant increase in immature metaphase 1 (M1) and germinal vesicle (GV) oocytes and deterioration of oocyte quality in patients with endometrioma greater than 3 cm in diameter [119, 120]. The recommended option for women with POF is to opt for IVF with donor oocytes. Patients with severely compromised immune systems are not recommended to undergo ovarian hyperstimulation for fertility preservation. Women and children with cancer suffer the most due to the significant impact of the malignancy on the ovarian reserve [80]. Cancer therapies such as chemotherapy and radiation can damage the tissue leading to a complete cessation of ovarian function. Since the ovaries are unresponsive to medical stimulation, assisted reproductive techniques cannot improve infertility [121]. Gestational surrogacy is one other option but is associated with legal, ethical, and economic problems [122].

### Hormone replacement therapy

One of the optimal strategies for primary ovarian failures is hormone replacement therapy (HRT) [123, 124]. HRT involves administering estrogen and progesterone to compensate for the hormonal imbalance, especially in post-menopausal women and POF cases [124]. It is an effective strategy for alleviating symptoms of hypoestrogenism and reducing the risk of cardiovascular diseases and osteoporosis. In addition, HRT can reduce the clinical complications of low estrogen levels and manage menopausal symptoms. Estrogen-based therapy post-menopause for managing night sweats, hot flashes, and vaginal dryness in women is associated with an increased risk of health issues [125]. They do not improve ovarian function or fertility, rather they may increase the risk of thrombotic disorders [126], endometrial [124] and breast cancer [127] in post-menopausal women [123].

### Tissue transplantation

Ovarian tissue cryopreservation, transplantation, oocyte, and embryo vitrification are the alternatives for fertility preservation in women and prepubertal girls diagnosed with cancer [128]. Life expectancy is significantly important for children and women diagnosed with critical illness. Even though the transplantation of ovarian tissue has resulted in live birth and initiation of puberty, there is only a restricted time limit during which the transplants can maintain normal hormone levels with menstrual cycles and have the potential to produce offspring [129]. Ovarian tissue cryopreservation has certain challenges and limitations causing impairment of ovarian microvasculature, oocyte DNA damage, tissue fibrosis, necrosis, ice crystal formation, and recrystallization during the thawing procedure can be damaging to the cells. The same applies to oocyte cryopreservation where the large volume of water in oocyte and ice crystal formation can damage the stability of actin microfilaments and microtubules responsible for cell motility and chromosome segregation [130]. However, tissue transplantation is not suitable for patients with ovarian cancer and leukemia due to the higher risk of reintroducing the carcinogenic material which could further worsen the condition [131–133].

## Innovative approaches for regenerating ovarian tissue

### Role of tissue engineering in treating ovarian disorders

Reproductive system diseases are becoming more common in women and younger children, and this has a serious negative impact on their reproductive health. Thus, the necessity for an efficient treatment technique is critical. Reproductive tissue engineering-based approach could replicate the complex architecture and hormone-influenced microenvironment during different stages of the reproductive cycle of the ovary [134]. Ovarian tissue engineering is continuously evolving as the aim is to mimic the microenvironment for germ cell development and reestablish fertility. It includes the combination of biomaterials, cells, and bioactive factors to facilitate tissue regeneration. A range of synthetic and natural biomaterials combination with various cell types have been used for the ovarian tissue regeneration process [12, 135]. Various growth factors stimulate the growth and development of these cells [136]. Reconstructing the ovarian microenvironment to restore the tissue integrity is a challenging task. Tissue engineering offers an advantage over the current treatment approach as sourcing of the materials and cells is less challenging. Reproductive tissue engineering has the potential to revolutionize women’s health [9]. The increase in cancer survivors makes tissue engineering an emerging strategy for follicular development and restoring fertility. There is also a lesser possibility of reintroducing the disease as compared to tissue transplantation [137]. The biomimetic models could be used as in-vitro models for drug analysis and in-vitro oocyte maturation in ART. Tissue-engineered scaffolds are highly preferred to restore the normal functioning of the organ [8]. Refinements and advancements in tissue engineering are being made to upgrade and standardize an effective technique for tissue regeneration.

### Role of nanotechnology in treating ovarian disorders

Reproductive medicine is a field that is constantly developing. It enables patients with acquired and congenital disorders associated with anatomic, genetic, and endocrine conditions to conceive and deliver, but the challenges remain in terms of the emotional and physical well-being of the patients throughout their lives. It is notable how reproductive tissue engineering has contributed to the field of reproductive medicine to mainly address the challenges associated with reproductive organs by restoring fertility, helping regenerate and replace tissues and organs efficiently for a longer period [136, 138]. The incorporation of nanotechnology with reproductive medicine has the potential to contribute to better patient care with advanced treatment of diseases [30, 31]. The possible combination of tissue engineering and nanomaterials could enhance the functionality of the biomimetic material and help enhance the cellular behaviour improving the functionality and development of engineered tissues and organs [139]. Nanotechnology has been successfully used in tissue engineering and regenerative medicine, drug delivery systems, early diagnosis, and targeted therapy [140]. To precisely target and treat disorders of the female reproductive system, nanomaterials can be functionalized and customized to reverse the disease pathology and reduce side effects. Many biocompatible materials have been used for targeting reproductive system disorders. Several strategies have been implemented with nanomaterials for monitoring, detection, and treatment of common reproductive disorders in women. These strategies include nano-therapy with vesicular lipid-based nanoparticles, gold and silver nanoparticles, and metal nanoparticles to treat reproductive system disorders [141]. Nano biosensors with silicon nanowires and nanocomposites with silver and graphene oxide have been developed for the detection of hormones like FSH and LH [142–144]. Improved viability and development rate of follicles were observed with in vitro culture of magnetic nanoparticles [145] and super magnetic iron oxide nanoparticles [146]. Addition of zinc nanoparticles with in vitro maturation media significantly increased the blastocyst rate of in vitro fertilized bovine oocytes [147]. Among all the different biocompatible nanoparticles, cerium oxide nanoparticles have gained significant attention with their diverse biomedical applications [148]. They are among the most promising nanoparticles because of their exceptional qualities and functional stability both in vitro and in vivo [149, 150]. Their autocatalytic antioxidant properties are beneficial in addressing ovarian disorders [32]. The current review highlights the antioxidant properties of CeO_2_ NPs and outlines the existing use of this nanomaterial for managing female reproductive disorders. Reproductive tissue engineering incorporating antioxidant nanomaterials to reduce disease pathophysiology could be a safe and sustainable alternative to address the challenges associated with current therapeutic methods [15, 27, 30].

### Role of cerium oxide nanoparticle in mitigating oxidative stress

Ceria is a highly prevalent and most abundant rare-earth metal, belonging to the lanthanide series on the periodic table found in the earth’s crust. In bulk state, they are found to exist in both tetravalent and trivalent stages, and their metal oxide stage exists as cerium dioxide (CeO_2_) and cerium sesquioxide (Ce_2_O_3_) [151]. Cerium is stable at the tetravalent stage as compared to the trivalent stage. At the nanoscale, they are observed to have a mixture of 4^+^ and 3^+^ states on the surface. As the particle diameter reduces the 3^+^ sites increase resulting in the loss of oxygen atoms on the surface of CeO_2_ NPs [149]. They can switch their oxidation state between the (^+^3) and (^+^4) stages and this redox behavior offers a multi-enzymatic activity enabling them to scavenge free radicles, mitigate oxidative stress, and protect against radiation [32, 152]. CeO_2_ NPs have become an increasingly important metal oxide nanoparticle primarily for their diverse application across various fields, especially in regenerative medicine and tissue engineering. They are observed to have anti-bacterial [153], antioxidant [154–156], self-renewing, autocatalytic [150], anti-inflammatory [157] and anti-cancer [158–160], angiogenic activities [161] making them suitable candidates for drug/gene delivery [162–164] and tissue engineering applications [149, 165, 166].

**Fig. 4:**
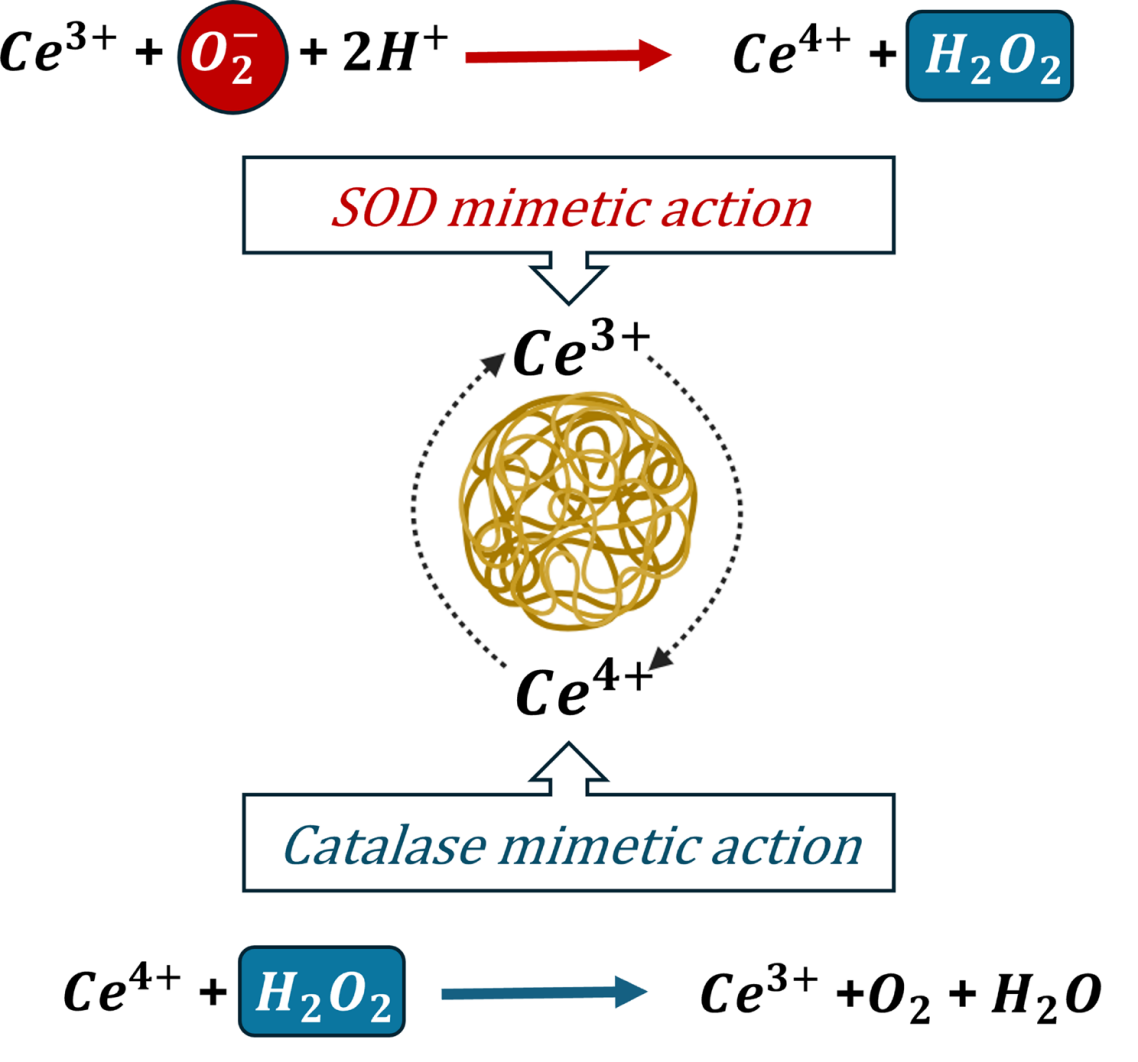
Enzyme mimetic action of nanoceria

CeO_2_ NPs have remarkable antioxidant potential. At neutral pH, they can mitigate the free radicals by acting as an antioxidant whereas, at acidic conditions, they can generate ROS at higher concentrations making them a pro-oxidant [151, 167]. The dual antioxidant and pro-oxidant nature of nanoceria makes them a suitable candidate for both in vitro and in vivo therapeutic and protective biomedical applications. At physiological pH, they neutralize free radicals in the cells and stabilize the ROS preventing oxidative damage, particularly in treating neurodegenerative and inflammatory diseases. Additionally, their enzyme mimetic action can modulate cellular antioxidant pathways enhancing the natural antioxidant defense system. Whereas, at lower acidic pH, longer exposure to high concentrations of nanoceria can kill cancer cells by generating ROS acting as a pro-oxidant. However, prolonged exposure of healthy cells to nanoparticles might alter the cellular structures as they interact with cell membrane proteins and mitochondria leading to ROS generation and oxidative stress. Depending on the physicochemical properties, method of synthesis, dose, type of target, concentration and exposure time nanoceria can have bioprotective (antioxidative), toxic (pro-oxidant), or no biological role [168]. Ceria in the nanoscale is observed to have a mixture of 3^+^ and 4^+^ states on the surface where they accept and donate electrons and participate in ROS scavenging [149]. They act by scavenging free radicals through the redox switch between Ce^4+^ and Ce^3+^ oxidation states where they can bind to oxygen reversibly and the cell response to nanoceria is dependent on the physicochemical properties like structure properties, particle size, concentration, and time-dependent based on various cells and tissues. They act by oxidation switch between the Ce^3+^ and Ce^4+^ stages depending on the environment. This removal and addition of oxygen atoms alter the surface of cerium atoms retaining the fluorite structure facilitating the autocatalytic regenerative property. CeO_2_ NPs function as both reducing and oxidation catalysts depending on the surrounding reactions. The high surface area and small particle size of nanoceria enhance their reactivity to free radicals, making them a potent antioxidant material for biomedical applications [29]. Their ability to switch between the oxidation states ubiquitously provides free radical scavenging properties comparable to biological antioxidants like superoxide dismutase (SOD) and catalase (Fig. 4).

SOD reacts with free radicals, mostly superoxide anions in the body, repairs and reduces cellular damage. SOD mimetic activity is superior with increased Ce 3^+^/Ce 4^+^ ratio. CeO_2_ NPs catalyze the dismutation of superoxide by binding to the oxygen vacancy site of Ce 3^+^, involving electron transfer to the oxygen atom and subsequent binding of two protons to the oxygen atom releasing H_2_O_2_. In the presence of a reducing agent like H_2_O_2_ the Ce 4^+^ is reduced to Ce 3^+^ after the transfer of electrons to cerium and the release of protons. Catalase is found in all organisms, when exposed to oxygen it acts as a protective enzyme against harmful oxidizing agents like H_2_O_2_. Catalase mimetic action of CeO_2_ NP is significantly dependent on increased levels of Ce 4^+^/ Ce 3^+^ ratio. The reaction occurs as follows, H_2_O_2_ reacts with Ce4 + and releases O_2_ and protons as well as reduces Ce 4 + to Ce 3+, and another reaction with H_2_O_2_ releases H_2_O and oxidizes Ce 3^+^ back to Ce 4^+^ state [149, 151]. The structural properties and paradoxical effect of CeO_2_ NPs enable both reduction and oxidation making them effective antioxidants. Both catalase and SOD enzyme mimetic actions must be coordinated along with proper pH, particle size, Ce 3^+^/Ce 4^+^ ratio for mitigation of free ROS. The auto-catalytic activity plays an important role in providing antioxidant action and is primarily dependent on the pH conditions. CeO_2_ NPs have regenerative properties only under basic physiological conditions and pH of 7.4 [150]. Oxidative stress plays a key role in the pathophysiology of most diseases, notably ovarian diseases. The radical ROS scavenging property of nanoceria is greatly beneficial in treating such disease conditions to protect organs and tissues from cellular damage caused by ROS and free radicals.

### Recent applications of cerium oxide nanoparticles for improving female reproductive function

**Fig. 5:**
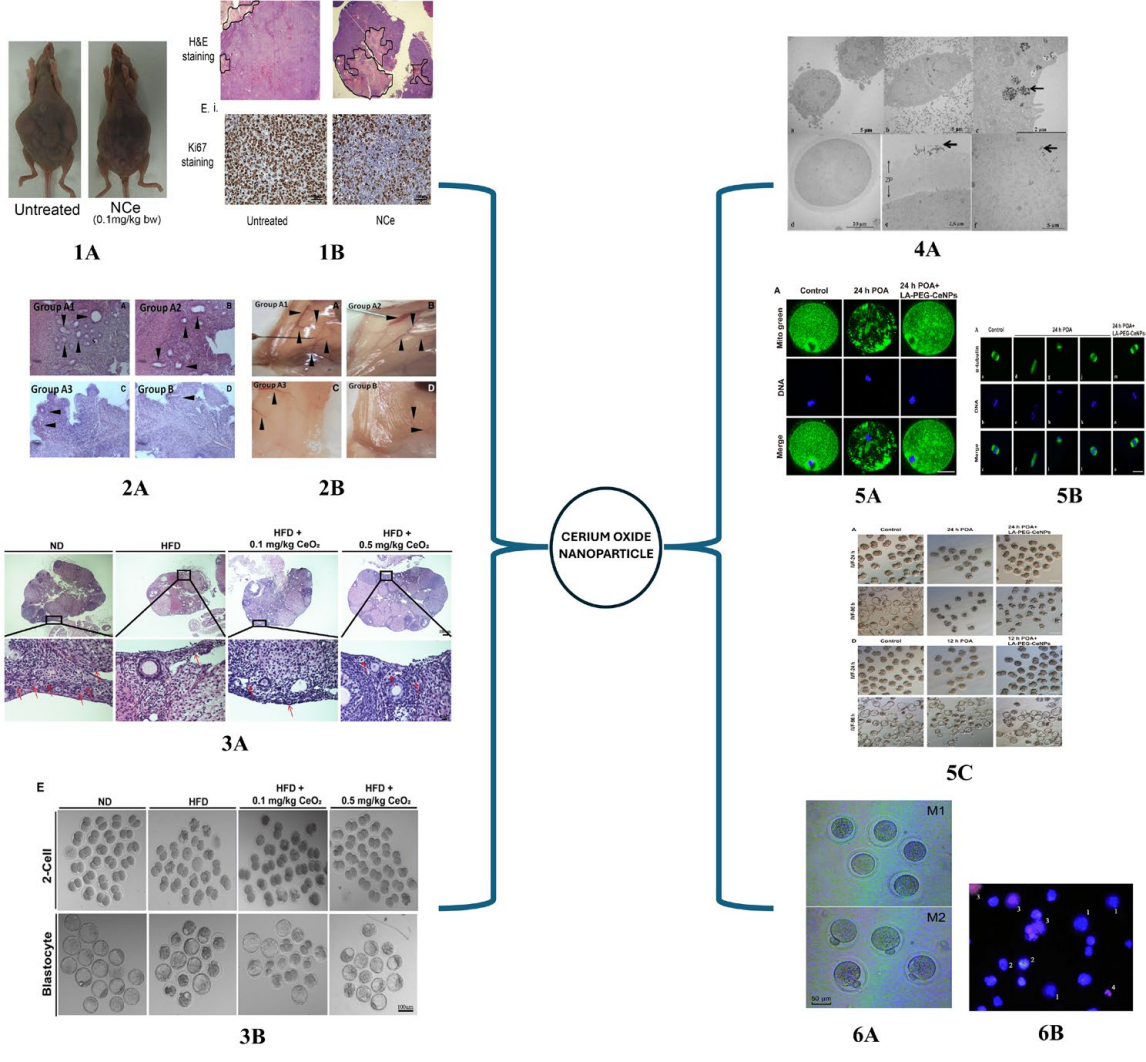
Application of cerium oxide nanoparticles for improving reproductive health. **1A**. Morphology of tumor induced mouse untreated and treated with nanoceria. **1B**. Representative H&E staining of treated and untreated xenografts with necrotic (pink) and live (purple) areas. (ii) Representative Ki-67 staining of xenografts [171] **2A**. Image of endometrial tissue H&E staining of endometriotic mice injected with (**A**) 0.9% NaCl (**B**) NAC (**C**) Nanoceria (**D**) control healthy mice with 0.9% NaCl. The arrow indicated endometrial glands. **2B**. Peritoneal cavity of endometriotic mice with similar treatment method and arrow indicates visible blood vessels [169] **3A**. Ovarian sections H&E images of mice fed with ND, HFD, HFD + CeO2 NPs at 0.1 mg/kg and 0.5 mg/kg. Enlarged images show the primordial follicles of each group. **3B**. Representative images of 2-cell and blastocyst embryos from each group [178] **4A**. Transmission electron microscopy images of follicles and oocytes exposed to CeO2 NPs. Arrow indicating their accumulation [172] **5A**. Representative image showing mitochondrial distribution in control, 24 h POA, LA-PEG-CeNPs stained using Mito Tracker Green. **5B**. The images of spindle morphology stained with Tubulin tracker green and chromosome alignment with Hoechst in each group. **5C**. Representative images to assess the fertilization and embryonic development showing the 2 cells and blastocyst developed from each group [180] **6A**. Light microscopy images of in vitro matured oocytes of nanoceria treated mice at M-I stage after 4 h culture and M-II stage after 20 h culture. **6B**. Fluorescent microscopy image of Hoechst 33,342 and propidium iodide-stained follicular cells: 1-viable cells, 2-apoptotic cells, 3-necrotic cells, 4-apoptotic bodies [170]

The successful use of CeO_2_ NPs for tackling reproductive tissue-related disorders to restore damaged ovarian function is summarized in Table 1. and Fig 5.

**Table 1:** Use of CeO_2_ NPs for improving female reproductive function

Nanoparticle	Model	Approach	Findings	Ref
CeO_2_ NPs	CD-1 strain Swiss Albino endometriosis female mice	Injecting 0.5 mg/kg nanoceria into the peritoneal cavity twice a day for 15 days	Mitigate endometrial lesions, decrease OS, inhibit angiogenesis, and increase oocyte quality.	[169]
CeO_2_ NPs	Old Balb/C and CBA mice	Treating old Balb/c mice with Ce0_2_ NPs at dose of 45 mg/kg once a day for 3 days	Antioxidant action, anti-aging effect, and activated meiotic maturation of oocytes with increased litter size in aged mice.	[170]
CeO_2_ NPs	Human ovarian cancer cell line SKOV3, HUVEC, A2780 injected mice, cancer model	In vitro culture with 50–100 µM nanoceria and in vivo intra-peritoneal injection of nanoceria 0.1 mg/kg every 3rd day for 30 days	In vivo and in vitro anti-angiogenic, metastasis inhibiting therapeutic role in ovarian cancer.	[171]
CeO_2_ NPs	Oocytes and follicular cells from old CD1 Female mice	In vitro maturation of oocytes and follicular cells in the presence of 2, 5, 10, and 100 mg/ml CeO_2_ NPs for 2 h	No internalization in oocyte cytoplasm, dose-dependent genotoxic effect.	[172]
CeO_2_ NPs	Ovine oocytes	In vitro maturation of oocytes in the presence of CeO_2_ NPs (0, 44, 88, 220 mg/mL)	No interference with nuclear and cytoplasmic maturation, increased blastocyst yield, and reduced ROS at the lowest dose.	[173]
CeO_2_ NPs	In vitro - OVCAR3, SKOV3 ovarian cancer cell line In vivo - A27890 generated mouse xenograft	24-hour incubation of cell lines with folic acid-tagged nanoceria NCe-FA (0- 300 µM) In vivo treatment with 0.1 mg/kg with NCe-FA alone and combined with cisplatinum for 3 days.	Enhanced specific targeting of ovarian cancer, apoptosis of tumor cells, inhibited ROS, cell proliferation, and migration.	[174]
CeO_2_ NPs	Human ovarian adenocarcinoma cell line - SKOV3	72-hour treatment with 50 µg/ml of 7 and 94 nm CeO_2_ NPs	No genotoxicity, ROS scavenging depends on the exposure time, size, pH, and cell type used.	[175]
CeO_2_ NPs	Pregnant Sprague Dawley rat	10 ml CeO_2_ NPs administered orally during pre-mating, mating, gestation, and early lactation periods with of 100, 300,100 mg/kg for 2 weeks	No biodistribution in rats or pubs. Excreted through feces in 24 h.	[176]
Cerium oxide CeO_2_	Pregnant NMRI mice	Intraperitoneal administration on days 7 and 14 of gestation with 10, 25, 80, and 250 mg/kg body weight of cerium oxide	Dose-dependent effect on primary and primordial follicles of the neonatal ovarian tissue	[177]
CeO_2_ NPs	High-fat diet (HFD) fed ICR female mice	HDF mice treated with 0.1 mg/kg and 0.5 mg/kg CeO_2_ NPs 3 times a week for 12 weeks	Restoration of glucose metabolism, oocyte, and blastocyst quality. Reduced endoplasmic reticulum and mitochondrial stress	[178]
CeO_2_ NPs	Dehydroepiandrosterone (DHEA) induced PCOD mouse model	Treated with CeO_2_ NPs chemically bonded with anti-inflammatory drug Resveratrol (CeO_2_@RSV) for 2 weeks	Mitigate excess ROS, inflammation, follicular atresia, and apoptosis of granulosa cells. Alter M1/M2 macrophage subtype	[179]
CeO_2_ NPs	Oocytes from female ICR mice	12/24-hour in vitro culture of oocytes with Lipolic acid (LA) and Polyethylene Glycol (PEG) modified CeO_2_NPs (LA-PEG-CeNPs)	ROS scavenging and multienzyme mimetic action, restore mitochondrial function, prevent post-ovulatory oocyte aging, and improve fertilization and embryo developmental potential.	[180]

CeO_2_ NPs have been successfully used for tackling reproductive tissue-related disorders in recent years [31] Chaudhury et al., identified the mitigation of endometriosis using regenerative cerium oxide nanoparticles. The study investigated nanoceria’s role in treating endometriosis associated with neovascularization and oxidative stress. Endometriosis, associated with high levels of ROS and lipid peroxidation rates and reduced antioxidant activity, is a disease with a high recurrence rate. In the study, CD-1 Swiss albino female mice were used and induced with endometriosis and injected with 0.5 mg/kg bodyweight nanoceria, 250 mg/kg N-acetyl cysteine, and 100 µl 0.9% NCl by dividing into 3 groups for 15 days. It was observed that nanoceria was most effective in reducing oxidative stress associated with endometriosis by reducing the ROS, lipid peroxidation rate, and increasing total antioxidant capacity. The three-fold reduction of adrenomedullin and vascular endothelial growth factor further confirmed the reduction in angiogenesis by nanoceria. There was also a pronounced reduction in the endometrial glands and micro vessel density observed in the morphometric analysis. They also identified a protective effect of nanoceria in the oocytes further confirming the clinical potential of nanoceria [169]. This protective effect of Ceria nanoparticles (CNs) on oocytes was further confirmed by Spivak et al. [170] who observed a positive influence of CNs on age-associated oxidative stress on the female gamete. The old Balb/c mice were treated once for 3 days with 45 mg/kg CNs and there was an increase in follicles and the number of MI and MII oocytes. The percentage of necrotic and apoptotic cells was reduced, and viable granulosa cells were increased in the presence of CNs. Oxidative stress plays a major role in modulating age-related infertility and weakens the antioxidant system. The failure of nuclear and cytoplasmic maturation in aged oocytes leads to a reduction in mature oocytes. The study observed CNs could activate the meiotic maturation of oocytes depending on the stage of the reproductive system, increase the rate of mature oocytes (MII) and litter size by enhancing the fertilization potential, and balance the production and inactivation of free radicals.

The concerns regarding the potential toxicity of nanoceria remain a paradox. The biological effect and interaction of CeO_2_ NPs with mice oocytes were investigated by Courbiere et al., [172]. The follicular cells and oocytes from mice with and without zona pellucida matured in vitro in the presence of 2, 5, 10, and 100 mg/L CeO_2_ NPs. It was observed that despite the presence or absence of zona pellucida the CeO_2_ NPs did not internalize into the oocyte cytoplasm. Even though there was dose-dependent DNA damage by CeO_2_ NPs on the germ cells particularly on oocytes without zona pellucida, at lower doses, zona pellucida trapping and follicular cell endocytosis could reduce the oxidative stress and DNA damage protecting the oocytes. Thus, confirming the physicochemical properties of the cell determines the genotoxicity of CeO_2_ NPs. This dose-dependent effect of CeO_2_ NPs was confirmed by Ariu et al., [173] who identified the effect of in vitro maturation of prepubertal sheep cumulus-oocyte complex in the presence of 0, 44, 88, and 220 µg/mL CeO_2_ NPs and assessed their developmental competence to preimplantation stage in vitro. The results were correlative to a previous study as the nanoparticle was restricted to somatic cells and there was no internalization to the oocyte cytoplasm or interfered with nuclear maturation of oocytes. They did observe a dose-dependent effect on the cytoskeleton structure, chromatin configuration, and F-actin assembly of matured oocytes. The lowest dose of 44 µg/mL had regular spindle patterns, increased blastocyst yield, higher inner cell mass, and trophectoderm cell number after in vitro fertilization. The lowest dose of nanoceria expressed downregulated levels of oxidative stress and apoptosis markers compared to other groups further confirming the potential application of low dose CeO_2_ NPs for in vitro maturation of oocytes with poor developmental competence.

Nanoceria was identified as a novel therapeutic anti-angiogenic agent in ovarian cancer by Giri et al., [171]. The major limitation of ovarian cancer, the most fatal gynecological malignancy, is that most patients are diagnosed at an advanced disease stage. Nanoceria, being an excellent antioxidant with regenerative ability makes them a potent therapeutic agent in ovarian cancer both in vivo and *in vitro.* The treatment of ovarian cancer cell lines (A2780, C200, SKOV3) with doses of 20–200 µM nanoceria could attenuate the oxidative stress and inhibit the migration and invasion of cancer cells although no effect was observed on cell proliferation *in vitro.* The nude mice model bearing A2780 ovarian carcinoma cell, when treated with the lowest dose of 0.1 mg/kg body weight showed a visible reduction in the tumor growth and micro-vessel density confirming the significant decrease in proliferation of cancer cells in the presence of nanoceria. During in vivo analysis, the study further confirmed the inhibition of metastasis and anti-angiogenic potential of nanoceria by targeting the apoptosis of endothelial cells. There was no abnormal physiology or cytotoxicity in vital organs in nanoceria-treated groups in vivo. In a later study by Hijaz et al., [174] nanoceria was tagged with folic acid (NCe-FA) to enhance the specificity for targeting ovarian cancer. They studied the in vitro and in vivo efficiency of folic acid-tagged nanoceria in combination with cisplatinum to inhibit ovarian cancer. NCe-FA conjugate had a dose-dependent effect on cells in vitro with a higher internalization rate thereby inhibiting tumor growth by initiating apoptosis. The ROS generation in the presence of conjugate was moderate in the acidic cancer environment but was significantly lower compared to NCe alone. The tumor size was reduced in the NCe-FA treated in vivo A2780 mice xenografts with no tissue necrosis or damage to any other vital organs. Tumor growth was inhibited with restricted angiogenesis, proliferation, enhanced apoptosis, and a moderate effect on ROS due to pH differences in the cancer microenvironment when a combination of cisplatinum with NCe-FA conjugate was used. A later study by Vassie et al., [175] further confirmed the pH-dependent ROS scavenging potential of CeO_2_ NPs. The study determined that the endocytosis of nanoparticles is dependent on the cell type and physicochemical properties of CeO_2_ NPs. The uptake of nanoceria on both ovarian and cancer cell lines is dependent on cell type, exposure, and size of the nanoparticle. It was shown that the ROS scavenging property of nanoceria varies between cell types and large-sized nanoceria had better ROS scavenging potential. These studies identify the feasibility and novel approach for using CeO_2_ NPs for targeted therapy of ovarian cancer.

The low dose of nanoceria had no adverse effect on the vital organs and was biocompatible. Lee et al., [176] identified no biodistribution of CeO_2_ NPs in the internal organs of rats or their pups. There were no adverse toxic effects in the reproductive and developmental function or general systemic function of Sprague Dawley rats even after repeated oral exposure to CeO_2_ NPs up to 1000 mg/kg during their pre-mating stage to lactating period. Assessments were done on the major organs like kidneys, liver, lungs, blood, and reproductive organs of both male and female rats and their pups. A marked toxicity was not identified in any internal organs and within 24 h, the orally administered CeO_2_ NPs were excreted through feces. The impact of CeO_2_ on the neonatal ovary during pregnancy was assessed by Nemati et al., [177] different doses of CeO_2_ (10, 25, 80, 250 mg/kg) were administered on the 7th and 14th day of gestation to pregnant female mice. The neonatal ovary collected 2 and 6 days postpartum was histologically examined and a dose-dependent effect was observed on the ovarian follicles of neonates. The biochemical analysis for oxidative stress markers 15 days post-partum on the blood serum of neonates showed no significant difference. The study observed a dose and time-dependent biphasic response of CeO_2_ at 10 mg/kg with a nonsignificant increase in primary follicles and lower oxidative stress.

Obesity is one of the major health issues associated with the current modern society and is detrimental to the offspring as well. This leads to obesity-induced ovarian-metabolic disorders, poor oocyte quality, and developmental potential due to increased oxidative stress making it challenging for ART. Yang et al., [178] discovered that CeO_2_ attenuated the ovarian dysfunction induced by a high-fat diet (HFD) in mice and suggested an alternative antioxidant therapy for obesity-induced female infertility. HFD-induced mice were treated with 0.1 mg/kg and 0.5 mg/kg CeO_2_ NPs and a dose-dependent accumulation was observed with no effect on viability of granulosa cells. They also observed that reduced blood glucose levels and lipid accumulation indicate restoration of normal metabolic functions by mitigating ROS. The meiotic defects in HFD-associated oocytes including spindle and chromosomal defects were reduced by CeO_2_ NPs. The endoplasmic reticulum stress was decreased in the presence of CeO_2_ NPs with improved mitochondrial function which had a positive influence on the quality of oocytes, embryo development potential, and blastocyst formation [125]. The successful treatment of the in vivo PCOS mouse model with CeO_2_ NPs bonded with the anti-inflammatory drug resveratrol (CeO_2_@RSV) reveals the exceptional clinical potential of CeO_2_ NPs to treat PCOS [179]. Proinflammation plays a significant role in disease progression. The CeO_2_@RSV could remodel the immune microenvironment of the ovary and manipulate the macrophages by promoting the M1 anti-inflammatory phenotype and reducing the M2 pro-inflammatory phenotype. Further, the stable antioxidant and anti-inflammatory activity combined with the polarization of macrophages of CeO_2_@RSV could yield the proliferation of granulosa cells and inhibit their apoptosis. Reversal of disease pathology was observed in the presence of nanocomposite as there was also the restoration of the ovarian morphology, endocrine function, and normal glucose metabolism. The study confirms the therapeutic ability of nanoparticles to manage chronic inflammatory PCOS and help restore ovarian function.

The potential clinical application of CeO_2_ NPs for ART was identified by Zhang et al., [180]. The mature MII oocytes undergo time-dependent degradation causing post-ovulatory aging and formation of low-quality oocytes particularly due to the imbalance in the antioxidant system. The excess ROS damages and degenerates the cellular functions. Post-ovulatory oocytes were treated with CeO_2_ NPs modified with lactic acid and polyethylene glycol (LA-PEG-CeNPs) for 12 and 24 h in vitro. In the presence of LA-PEG-CeNPs, there was a significant reduction in oocyte apoptosis and fragmentation thereby increasing the quality of oocytes. The nanoparticle was effective in mitigating the excess ROS with their catalase and SOD mimicking action, restoring mitochondrial equilibrium and function, which further balanced the ATP production. This further rescued the oocyte morphology by reducing the fragmentation rates and enhancing their developmental potential. The spindle organization and chromosomal alignment play a major role in the quality of oocytes, and the restoration of physiological ROS could attenuate related abnormalities. The abnormal spindle promotes unequal separation of chromosomes and in the presence of LA-PEG-CeNPs, there was normal chromosome segregation with partial restoration of filamentous actin. The study also investigated the fertilization and embryo developmental potential of LA-PEG-CeNPs treated oocytes. After IVF there was pronounced improvement in the rate of fertilization and embryo development of 12-hour LA-PEG-CeNPs treated oocytes as compared to 24-hour treated oocytes. The results suggest the protective effect of LA-PEG-CeNPs on aged oocytes with reduced oxidative damage, improving their fertilization and blastocyst formation rates. This further confirms the potential clinical application of nanoparticles in ART. Literature findings suggested the biocompatibility, bioavailability, and excellent ROS scavenging potential of CeO_2_ NPs and their therapeutic role in reversing the disease pathophysiology. The novel application of nanotechnology in reproductive tissue engineering is still in its infancy and could be explored to develop efficient tissue regeneration techniques.

## Conclusion and future perspectives

Infertility has become a prevalent condition in present society worldwide. There is a significant rise in the rate of infertility with advancing age, socioeconomic status, and reproductive system disorders contributing to delayed childbirth. Although male infertility contributes to challenges in conception, female infertility remains the primary cause. Female infertility continues to be a taboo in the present day society. Reproductive health has a direct impact on the quality of life and fertility status in women. It is a primary factor in determining a women’s life span. The current treatment options are limited and frequently fail to restore tissue function, necessitating alternative treatment strategies. Throughout the years the development of regenerative medicine and tissue engineering have contributed to improving female reproductive health. The ovaries age more rapidly than other body systems, showing a steady decline in both the quantity and quality of female gametes. Ovarian disorders have an alarming deleterious effect on the female reproductive system. This review is focused on addressing multifactorial complex ovarian diseases and identifying an innovative approach to restore fertility. The current treatment options are inadequate for meeting the standard requirements for restoring fertility and endocrine functions. Reproductive tissue engineering has emerged to be a significant treatment option. The review outlines the use of nanotechnology and its potential combination with tissue engineering as an advanced option for improving tissue regeneration in reproductive diseases. While complex pathologies undoubtedly contribute to ovarian diseases, there is growing attention to the significant impact of oxidative stress. The health of ovaries influences the well-being of women and children at every stage of life. This review specifically highlights CeO_2_ NPs and their role in mitigating the oxidative stress associated with ovarian diseases. CeO_2_ NPs are observed to be excellent nanoparticles in restoring the normal physiology of the organ by effectively improving oocyte quality, fertilization potential, and rate of blastocyst development. Furthermore, these studies confirm the biocompatibility of CeO_2_ NPs and their potential clinical significance by reversing the disease pathologies. The review highlights the potential combination of nanotechnology with reproductive tissue engineering for an effective treatment strategy to address infertility and its related challenges in women.

## Data Availability

No datasets were generated or analysed during the current study.
